# Facilitation by intracellular carbonic anhydrase of Na^+^–HCO_3_^−^ co-transport but not Na^+^/H^+^ exchange activity in the mammalian ventricular myocyte

**DOI:** 10.1113/jphysiol.2013.265439

**Published:** 2014-01-09

**Authors:** Francisco C Villafuerte, Pawel Swietach, Jae-Boum Youm, Kerrie Ford, Rosa Cardenas, Claudiu T Supuran, Philip M Cobden, Mala Rohling, Richard D Vaughan-Jones

**Affiliations:** 1 Burdon Sanderson Cardiac Science Centre, Department of Physiology, Anatomy and Genetics, University of OxfordOxford OX1 3PT, UK; 2 Department of Physiology and Biophysics, College of Medicine, Cardiovascular and Metabolic Disease Center, Inje UniversityBusan 614-735, Korea; 3 Università degli Studi di Firenze, Dipartimento di Scienze farmaceutiche50019 Florence, Italy

## Abstract

Carbonic anhydrase enzymes (CAs) catalyse the reversible hydration of CO_2_ to H^+^ and HCO_3_^−^ ions. This catalysis is proposed to be harnessed by acid/base transporters, to facilitate their transmembrane flux activity, either through direct protein–protein binding (a ‘transport metabolon’) or local functional interaction. Flux facilitation has previously been investigated by heterologous co-expression of relevant proteins in host cell lines/oocytes. Here, we examine the influence of intrinsic CA activity on membrane HCO_3_^−^ or H^+^ transport via the native acid-extruding proteins, Na^+^–HCO_3_^−^ cotransport (NBC) and Na^+^/H^+^ exchange (NHE), expressed in enzymically isolated mammalian ventricular myocytes. Effects of intracellular and extracellular (exofacial) CA (CA_i_ and CA_e_) are distinguished using membrane-permeant and –impermeant pharmacological CA inhibitors, while measuring transporter activity in the intact cell using pH and Na^+^ fluorophores. We find that NBC, but not NHE flux is enhanced by catalytic CA activity, with facilitation being confined to CA_i_ activity alone. Results are quantitatively consistent with a model where CA_i_ catalyses local H^+^ ion *delivery* to the NBC protein, assisting the subsequent (uncatalysed) protonation and removal of imported HCO_3_^−^ ions. In well-superfused myocytes, exofacial CA activity is superfluous, most likely because extracellular CO_2_/HCO_3_^−^ buffer is clamped at equilibrium. The CA_i_ insensitivity of NHE flux suggests that, in the native cell, intrinsic mobile buffer-shuttles supply sufficient intracellular H^+^ ions to this transporter, while intrinsic buffer access to NBC proteins is restricted. Our results demonstrate a selective CA facilitation of acid/base transporters in the ventricular myocyte, implying a specific role for the intracellular enzyme in HCO_3_^−^ transport, and hence pH_i_ regulation in the heart.

## Introduction

Carbonic anhydrase (CA) proteins are expression products of a gene-family that codes for at least 16 isoforms (Sly & Hu, 1995; Supuran, 2008). All except CAVII, CAX and CAXI are functionally active enzymes, widely expressed, which catalyse the reversible hydration of CO_2_ to H^+^ and HCO_3_^−^ ions. Catalytically active CAs fulfil a number of functions (Maren, 1967). They can accelerate the cellular venting of CO_2_ (Geers & Gros, 2000; Swietach *et al*. 2008, 2009), the spatial diffusion of intracellular and extracellular H^+^ ions (Stewart *et al*. 1999; Spitzer *et al*. 2002; Swietach *et al*. 2009; Hulikova *et al*. 2011), and the speed of CO_2_/HCO_3_^−^ (carbonic) buffering (Leem & Vaughan-Jones, 1998). A major role for CAs is proposed to be the catalytic enhancement of acid/base membrane transport. Some reports suggest they achieve this by binding directly to pH regulatory transporters, forming a multimeric protein complex called a transport metabolon (Sterling *et al*. 2001, 2002; Morgan *et al*. 2007; Casey *et al*. 2009; Svastova *et al*. 2012; Vargas & Alvarez, 2012). The proposal remains controversial, as observations of direct CA binding and flux facilitation have recently been challenged (Boron, 2010; Al-Samir *et al*. 2013; Parker & Boron, 2013), while the molecular mechanisms involved are far from clear. In some cases, for example, CA protein may stimulate acid transport allosterically, even in the absence of enzymic activity (Becker *et al*. 2011).

Various CA isoforms are expressed in mammalian heart. In ventricular myocytes, CAII is a cytosolic soluble intracellular isoform, CAIV and XIV are sarcolemmally tethered with exofacially exposed catalytic sites, while CAXIV and IX have also been detected at sarcoplasmic reticular and possibly transverse tubule membranes (Scheibe *et al*. 2006; Schroeder *et al*. 2013). CA activity in these cells has been shown to accelerate regulatory pH_i_ recovery following intracellular acid or base loads (Lagadic-Gossmann *et al*. 1992; Orlowski *et al*. 2011; Vargas & Alvarez, 2012). Moreover, the principal acid-extruding transporters in these cells, Na^+^/H^+^ exchange (NHE1 isoform) and Na^+^–HCO_3_^−^ cotransport (NBCe1 and NBCn1) (Aiello *et al*. 1998; Yamamoto *et al*. 2005; De Giusti *et al*. 2011; Garciarena *et al*. 2013; for review see Vaughan-Jones *et al*. 2009), when co-expressed heterologously with intracellular CAII or exofacial CAIV in oocytes or cultured cell lines, exhibit enhanced activity that is slowed in the presence of pharmacological CA inhibitors such as acetazolamide (ATZ) (Gross *et al*. 2002; Li *et al*. 2002; Alvarez *et al*. 2003; Loiselle *et al*. 2004; Li *et al*. 2006; Becker & Deitmer, 2007; Schueler *et al*. 2011). It has yet to be shown, however, that NHE and NBC activities are specifically regulated by either intracellular or exofacial CA in native ventricular cells.

In the present work we have examined the influence of the catalytic activity of intracellular and exofacial CA (CA_i_ and CA_e_, respectively) on NHE-and NBC-mediated acid efflux from rat ventricular myocytes. Intrinsic transporter and CA activity in these cells has been assessed from measurements of intracellular pH, intracellular [Na^+^], and extracellular surface pH (pH_e_) using ion-selective fluorophores (Leem *et al*. 1999; Yamamoto *et al*. 2005; Schroeder *et al*. 2013), while applying membrane-permeant and –impermeant CA inhibitors to gauge the relative importance of CA_i_ and CA_e_. Results suggest a novel and selective role for intracellular catalytic CA activity in controlling membrane acid extrusion, while questioning any obligatory role for exofacial CA. The results have important implications for the cytoplasmic organization of spatial H^+^ ion movement in the native ventricular myocyte.

## Methods

### Solutions

Lysis buffer contained: 15 mm NaCl, 105 mm potassium gluconate, 35 mm KCl, 20 mm Mes, 20 mm Hepes, 1 mm MgCl_2_, 0.5 mm EGTA; pH adjusted to 8.1 at 4°C with 5N HCl and 4M KOH. Hepes-buffered Tyrode solution contained: 135 mm NaCl, 20 mm Hepes, 4.5 mm KCl, 1 mm MgCl_2_, 2 mm CaCl_2_, 11 mm glucose; pH adjusted to 7.4 at 37°C with 4 m NaOH. CO_2_/HCO_3_^−^-buffered Tyrode solution contained: 125 mm NaCl, 4.5 mm KCl, 1 mm MgCl_2_, 2 mm CaCl_2_, 11 mm glucose, 22 mm NaHCO_3_ (or 12 mm NaHCO_3_ for surface pH measurements); bubbled with 5% CO_2_–95% O_2_ to adjust pH to 7.4 at 37°C. High-K^+^ CO_2_/HCO_3_^−^-buffered Tyrode solution contained: 85 mm NaCl, 45 mm KCl, 1 mm MgCl_2_, 2 mm CaCl_2_, 11 mm glucose, 22 mm NaHCO_3_; bubbled with 5% CO_2_–95% O_2_ to adjust pH to 7.4 at 37°C. Ammonium-containing solutions contained NaCl isosmotically replaced with NH_4_Cl. Superfusion solutions were delivered to the superfusion chamber at 2 ml min^–1^, 37°C.

### Drugs

#### NHE and NBC inhibitors

Cariporide (NHE inhibitor) and S0859 (NBC inhibitor) were kindly provided by Sanofi Aventis (Germany). Dimethylamiloride (DMA; NHE inhibitor) was purchased from Sigma (Poole, UK).

#### CA inhibitors

Membrane-permeable acetazolamide (ATZ) and ethoxyzolamide (ETZ) were obtained from Sigma. ATZ and ETZ block both intracellular and exofacial CA isoforms. Membrane-impermeant compound 14v (1-[5-sulfamoyl-1,3,4-thiazol-2-yl-(aminosulfonyl-4-phenyl)]-2,6-dimethyl-4-phenyl-pyridinium perchlorate; Alvarez *et al*. 2003; Supuran *et al*. 2004) is a positively charged heterocyclic sulphonamide that inhibits extracellular CA isoforms with nanomolar efficacy (Scozzafava *et al*. 2000; Supuran *et al*. 2004).

### Isolated rat ventricular myocytes

Male Sprague–Dawley rats (300–400 g) were killed by cervical dislocation in accordance with Home Office regulations for Schedule 1 methods of killing, and guidelines issued by the University of Oxford. Ventricular myocytes were enzymically dispersed from Langendorff-perfused hearts *in vitro* as described previously (Garciarena *et al*. 2013), using a mixture of protease and collagenase type II enzymes (Worthington), or Liberase (Roche Pharmaceuticals). Isolated cells were superfused with Hepes or CO_2_/HCO_3_^−^-buffered Tyrode solutions at 37°C. Quiescent, Ca^2+^-tolerant, rod-shaped cells were selected for experiments.

### *In vitro* carbonic anhydrase activity assay

Whole rat ventricles were dissected from Langendorff-perfused hearts *in vitro*. They were were homogenized in lysis buffer on ice for 3 min using a Polytron tip. Membranes were disrupted by a repeated freeze–thaw cycle to release all intracellular CA isoforms (Schroeder *et al*. 2013). For the best results, samples were diluted up to a total protein concentration of between 1 and 10 mg ml^−1^ (measured by the Bradford assay). Concentrations within this range give adequate enzyme concentration for a reliable CA measurement, without exceeding the resolving power of the measurement system. A 0.67 ml volume of lysate was added to the 2 ml capacity stirred chamber to which a pH electrode (Hamilton Biotrode Fisher Scientific, UK) was inserted to measure medium pH continuously at 1 Hz. The chamber was cooled to 4°C. To initiate the CA-catalysed reaction, 0.33 ml of CO_2_-saturated water (at 4°C) was added rapidly. The rate of acidification, measured by the electrode, was fitted using a least-squares algorithm to derive a hydration rate constant. The obtained rate constants were normalized to spontaneous rates measured in the presence of 100 μm ATZ, measured for the same dilution of lysate. As a result of this normalization procedure, enzyme concentration is eliminated as a variable.

### Fluorescence measurements

Intracellular pH, intracellular [Na^+^] and surface extracellular pH were measured using fluorescent indicators cSNARF1, SBFI (Yamamoto *et al*. 2005) and wheat germ agglutinin (WGA)-conjugated fluorescein (Stock *et al*. 2007; Schroeder *et al*. 2013) (Invitrogen, UK). Myocytes were loaded with fluorescein dyes (10 min acetoxymethyl ester (AM)-loading, at room temperature, with 10 μm cSNARF1; 60 min AM-loading with 100 μm SBFI; 3 min loading with 10 μm WGA-fluorescein) and allowed to settle at the base of a Perspex superfusion chamber mounted on an epifluorescence setup (Nikon Diaphot 200 microscope (Nikon, UK) with a Cairn monochromator light source (Cairn, UK) and Electron Tubes photomultipliers (Electron Tubes, UK) connected via an in-house designed side-arm) for measurements with cSNARF1 or SBFI, or a confocal fluorescence setup (LSM700, Zeiss, UK) for measurements with WGA-fluorescein. cSNARF1 was excited at 540 ± 5 nm and fluorescence was measured simultaneously at 640 ± 10 nm and 580 ± 10 nm by separating emitted wavelengths using a 605 nm dichroic mirror. SBFI excitation was alternated every 0.25 s between 350 ± 5 nm and 380 ± 5 nm, and the fluorescence emission was measured above 510 nm using a long-pass filter. WGA-fluorescein was excited by a 488 nm laser-line and fluorescence emission was measured above 510 nm using a long-pass filter.

Intracellular ratiometric fluorescence signals were calibrated *in situ* as previously described (Leem *et al*. 1999; Yamamoto *et al*. 2005; Schroeder *et al*. 2013), using high-K^+^, nigericin-containing superfusates of different pH (for intracellular cSNARF1 signal), superfusates of different pH (for extracellular surface WGA-fluorescein signal), and superfusates of different Na^+^ concentration containing the cation ionophores gramicidin and monensin (for intracellular SBFI signal). The intracellular SBFI signal recorded during an experiment shows a modest pH_i_ sensitivity (Minta & Tsien, 1989). This was corrected for by recording a pH_i_ time course (cSNARF1; not shown) in parallel experiments, and then applying the equation: [Na^+^]_corr_ = [Na^+^]_uncorr_ – α(pH_i_ – 7.2), to the [Na^+^]_i_ time course, where [Na^+^]_uncorr_ is the uncorrected calibrated signal, [Na^+^]_corr_ is the corrected signal, and the best-fitted value for the coefficient, α, was 3.0 (Yamamoto *et al*. 2005).

### Calculating acid flux

Acid efflux on NHE or NBC (or both) was calculated as the product of the rate of pH_i_ recovery from an acid load (dpH_i_/d*t*; piecewise fitted to a straight line over a 3 s interval) and buffering capacity (β). For experiments performed in the absence of CO_2_/HCO_3_^−^, β was equal to the intrinsic buffering capacity measured previously (Zaniboni *et al*. 2003). For experiments performed in the presence of CO_2_/HCO_3_^−^, the additional CO_2_-dependent component to β was calculated using the equilibrium equation 2.303 × [HCO_3_^−^]_o_ × 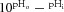 (Leem *et al*. 1999). The same calculation was performed to derive flux from mathematically simulated pH_i_ time courses.

### Statistics

Data are reported as mean ± standard error of the mean (SEM). Statistical significance between data was assessed by means of Student's unpaired *t* test.

## Results

### Catalytic activity of cardiac carbonic anhydrase

#### Intracellular and extracellular CA

Cardiac homogenization, by lysing cell membranes, exposes both intracellular and extracellular CA activity. Figure 1*A* shows that addition of 0.33 ml CO_2_-saturated water to 0.67 ml of buffer solution containing homogenized ventricular tissue reduced its pH over a period of 300 s, while addition of CA inhibitors ATZ (100 μm) or 14v (100 nm) slowed the reaction to a rate seen in the absence of homogenate. The rate constant for CO_2_ hydration (*k*_f_) in cardiac homogenate was reduced 10-fold by the inhibitors, while the residual rate constant equalled that for the spontaneous, uncatalysed reaction at 4°C (Fig. 1*B*). Ventricular tissue therefore expresses significant CA catalytic activity, as reported previously. In further experiments (not shown), the IC_50_ values for cardiac CA inhibition by ATZ and 14v were 7.3 ± 1.0 nm and 2.4 ± 0.6 nm, respectively (*n* = 10).

**Figure 1:**
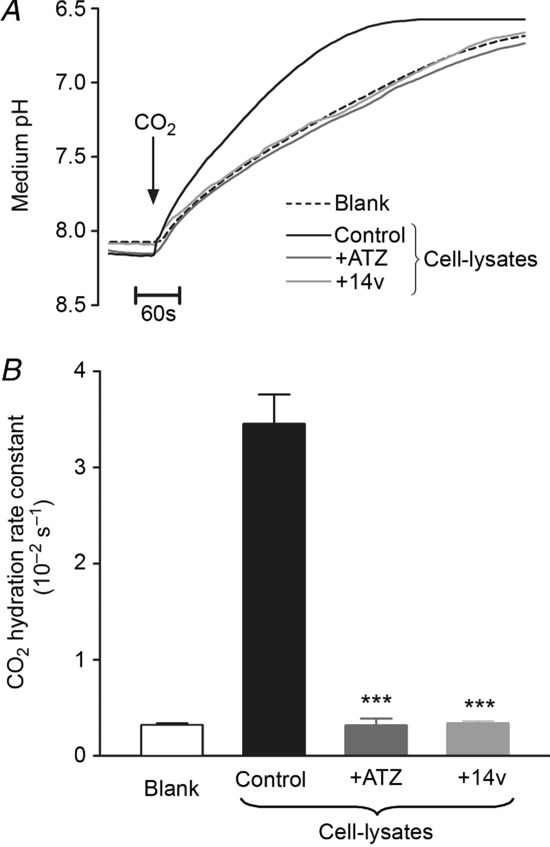
*A*, time course of medium pH measured at 4°C. Addition of CO_2_-saturated water to cardiac homogenate (prepared in lysis buffer) reduces medium pH at a rate proportional to CA activity. ATZ (100 μm) or 14v (100 nm) reduced the rate of acidification to spontaneous levels (i.e. measured in lysis buffer only). *B*, CA activity, expressed as the CO_2_ hydration constant *k*_f_, was significantly reduced in the presence 100 μm ATZ or 100 nm 14v to the level measured in ‘blank’ lysis buffer (*n* = 10 each). ^***^P < 0.001.

#### Intracellular CA

In intact cells, intracellular CA activity was assessed from intracellular cSNARF1 measurements of intracellular pH (pH_i_). Figure 2*A* shows that pH_i_ of an isolated myocyte rapidly acidified during superfusion of a 5% CO_2_/HCO_3_^−^-buffered Tyrode solution (CO_2_ is membrane permeant and hydrates within the cytoplasmic compartment). This acidification was slowed by adding 100 μm ATZ (Fig. 2*A* and *B*i). The suprathreshold dose, similar to that used previously (Schroeder *et al*. 2013), ensured adequate membrane permeation, thus leading to inhibition of both CA_e_ and CA_i_. The drug also slowed the subsequent rapid alkalinization of pH_i_ upon CO_2_ removal (Fig. 2*A* and *B*ii). In contrast, the membrane-impermeant CA inhibitor, 14v, had no effect on the pH_i_ changes (Fig. 2*B*), consistent with its inability to enter the cell. Figure 2*C* pools data from several experiments, and shows that the intracellular CO_2_ hydration constant (*k*_f_) was reduced about 3–fold by ATZ, as reported previously (Leem & Vaughan-Jones, 1998; Schroeder *et al*. 2013), but was unaffected by 14v.

**Figure 2:**
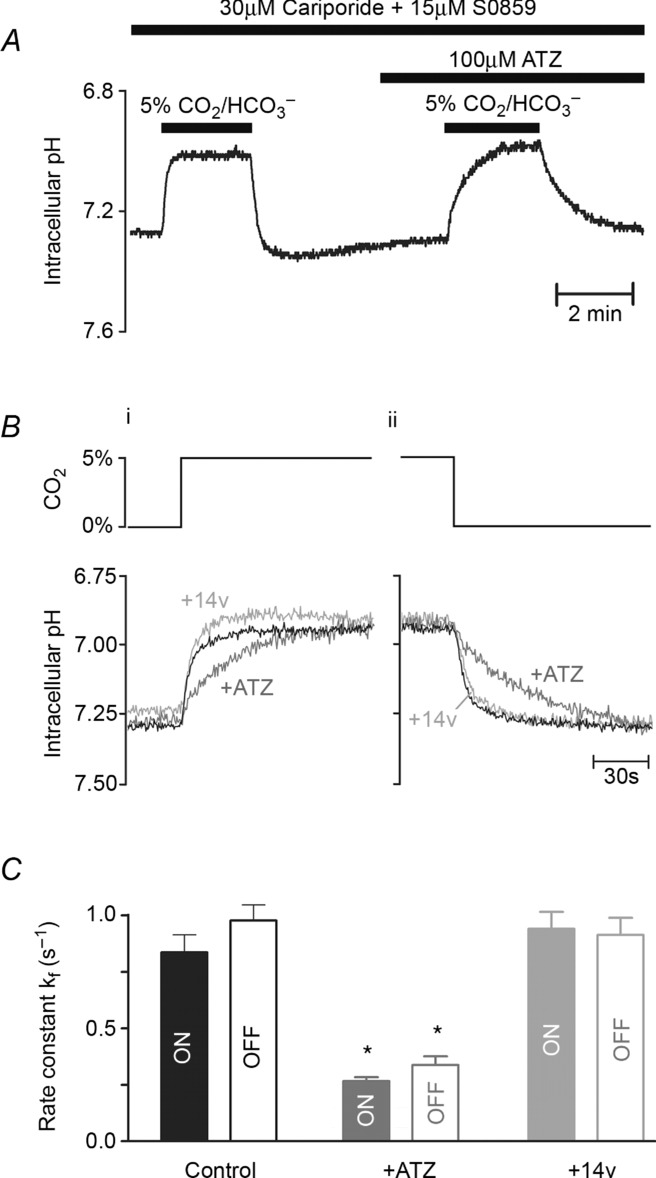
*A*, time course of intracellular pH (pH_i_; AM-loaded cSNARF1 fluorescence) during rapid solution switching between Hepes-buffered solution (CO_2_ free) and 5% CO_2_/22 mm HCO_3_^−^-buffered solution at 37°C (solutions included 30 μm cariporide and 15 μm S0859 to block acid extrusion on Na^+^/H^+^ exchange and Na^+^–HCO_3_^−^ co-transport, respectively). The initial rate of pH_i_ change is proportional to the intracellular CA activity. Paired experiment in the presence of ATZ to block CA activity. *B*, details of pH_i_ time courses measured upon switching from Hepes-buffered to CO_2_/HCO_3_^−^-buffered solution (i) or the reverse solution change (ii) under control conditions (black), in the presence of 100 μm ATZ (dark grey) or 100 nm 14v (light grey). *C*, CO_2_ hydration constant *k*_f_ derived from the initial rate of pH_i_ change upon CO_2_/HCO_3_^−^ addition (filled bars) or removal (open bars) under control conditions (*n* = 15) or in the presence of ATZ (*n* = 15) or 14v (*n* = 15). *P < 0.05.

#### Extracellular CA

In isolated myocytes, an exofacial, membrane-tethered CA enzyme is also catalytically active. This is illustrated in Fig. 3*A*, which shows extracellular surface pH (pH_e_) recorded in the isolated ventricular myocyte, obtained with the membrane-tagging pH fluorophore, wheat germ agglutinin-conjugated fluorescein (WGA-fluorescein). Superfusion of 30 mm NH_4_Cl induced a large transient acidification of pH_e_. This is caused by rapid, passive permeation of NH_3_ molecules into the cell, thereby promoting dissociation of local extracellular NH_4_^+^ into NH_3_ and H^+^, which reduces pH_e_. In CO_2_/HCO_3_^−^-buffered superfusates, the H^+^ ions liberated at the extracellular surface are partly absorbed by extracellular carbonic buffer, as illustrated schematically in the inset diagram. Inhibiting CA_e_ with ATZ would be expected to slow this buffering reaction thus rendering it less effective, and it is notable that in the presence of the drug, the transient fall of pH_e_ became considerably larger. Subsequent removal of extracellular NH_4_Cl produced transient changes of pH_e_, this time in the alkaline direction, as NH_3_ molecules fluxing out of the cell combined locally with extracellular H^+^. The pH_e_ transient was again enhanced in the presence of ATZ. In seven experiments, the amplitude of the pH_e_ transients was increased in the presence of ATZ by 68 ± 9% and 77 ± 20% on addition and removal, respectively, of NH_4_Cl (Fig. 3*B*). Results are thus consistent with inhibition by ATZ of CA activity expressed at the extracellular surface of isolated myocytes.

**Figure 3:**
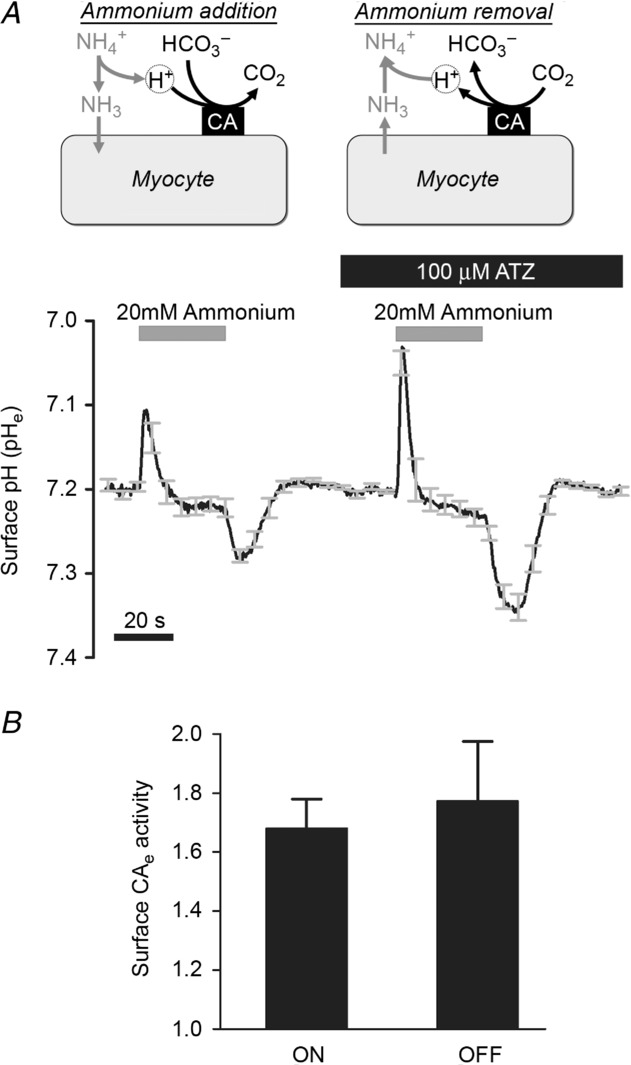
*A*, time course of extracellular surface pH (pH_o_; membrane-loaded wheat germ agglutinin-tagged fluorescein) during rapid and transient exposure to 30 mm NH_4_^+^/NH_3_ in 5% CO_2_/12 mm HCO_3_^−^-buffered solutions at 37°C (average of 9 cells). Exposure to NH_4_^+^/NH_3_ triggers surface acidification due to NH_3_ entry; subsequent withdrawal of NH_4_^+^/NH_3_ drives surface alkalinization. The amplitude of pH_e_ changes depends on CO_2_/HCO_3_^−^ buffering kinetics, catalysed by exofacial CA (CA_e_) activity. Paired experiment in ATZ to inhibit CA activity. *B*, CA_e_ activity quantified as a ratio of pH_e_ transient amplitude measured in ATZ and in control conditions for the addition (ON) and removal (OFF) of NH_4_^+^/NH_3_ (*n* = 9).

We conclude that CA is functionally active in the intracellular cytoplasmic and the extracellular exofacial domains of the myocyte.

### NHE activity unaffected by CA_i_ or CA_e_

Various protocols were used to assess NHE activity from pH_i_ measurements in isolated myocytes. Acid extrusion was stimulated by prepulsing a cell with 20 mm NH_4_Cl to reduce pH_i_. When using Hepes-buffered superfusates, NHE activity exclusively mediates the subsequent pH_i_ recovery to more alkaline levels (Fig. 4*A*), while a simultaneous acid efflux on NBC contributes when CO_2_/HCO_3_^−^ superfusates are employed (Fig. 4*B*). Under these latter conditions, the component due to NBC can be revealed by selectively inhibiting NHE, e.g. with 30 μm DMA (Fig. 4*C*). The relative contributions of NHE and NBC to acid efflux in the presence of CO_2_/HCO_3_^−^ buffer have been computed in Fig. 4*D*. At low pH_i_, acid extrusion rises steeply, with the larger efflux contributed by NHE. At less acidic levels of pH_i_, both NHE and NBC contribute about equally, as reported previously in ventricular myocytes (Leem *et al*. 1999). Estimates of acid extrusion into Hepes media, and DMA-sensitive extrusion into CO_2_/HCO_3_^−^-buffered media, were then used to compare NHE activity in the absence and presence of CO_2_/HCO_3_^−^ buffer, as plotted in Fig. 4*E*. At any given pH_i_, the two fluxes are virtually identical suggesting that CA, which is likely to be functional only in the presence of CO_2_/HCO_3_^−^, does not directly catalyse the activity of NHE. This result conflicts with previous reports of NHE/CA interaction in heterologous expression systems (Li *et al*. 2002, 2006), where CO_2_/HCO_3_^−^ buffering in combination with intracellular CA catalytic activity appeared to enhance NHE flux.

**Figure 4:**
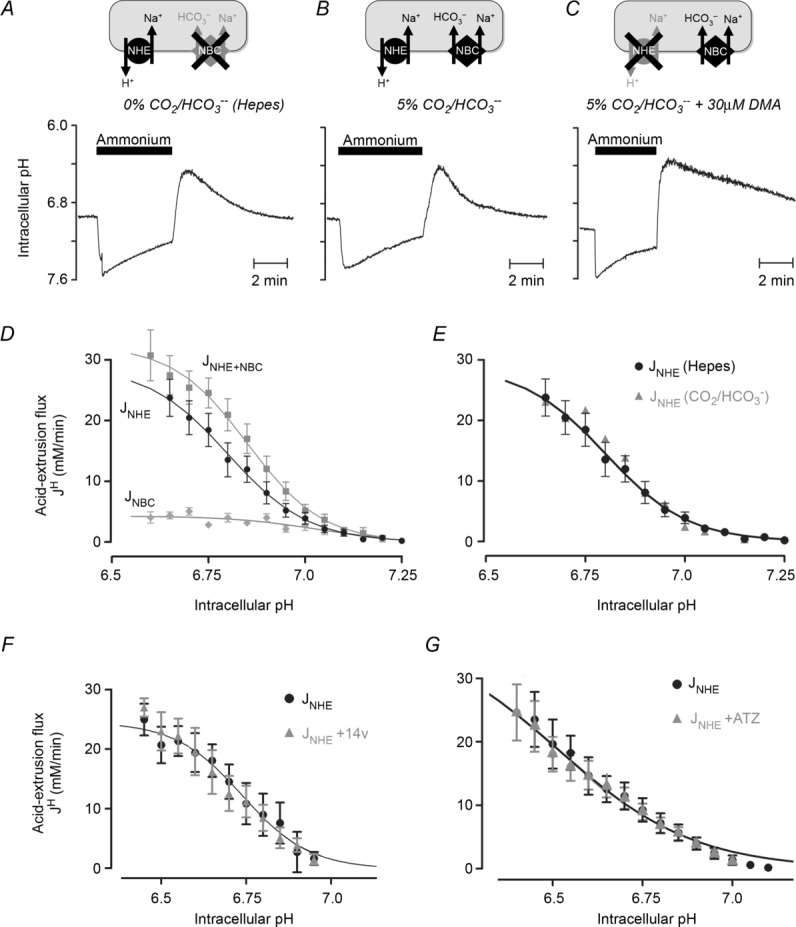
*A*, time course of intracellular pH during 20 mm ammonium prepulse solution manoeuvre. Recovery of pH_i_ from acid load in the absence of CO_2_/HCO_3_^−^ is mediated by Na^+^/H^+^ exchange (NHE). *B*, recovery of pH_i_ in the presence of CO_2_/HCO_3_^−^ is mediated by Na^+^/H^+^ exchange (NHE) and Na^+^–HCO_3_^−^ co-transport (NBC). *C*, recovery of pH_i_ in the presence of CO_2_/HCO_3_^−^ and NHE inhibitor dimethylamiloride (DMA) is mediated by NBC only. *D*, pH_i_ dependence of NHE-mediated acid efflux (*J*_NHE_; measured in Hepes buffer), NBC-mediated acid efflux (*J*_NBC_; measured in CO_2_/HCO_3_^−^ buffer and DMA) and the sum of NHE and NBC fluxes (*J*_NHE+NBC_; measured in CO_2_/HCO_3_^−^ buffer). Each data-point is the mean of 10–20 cells. *E*, flux analysis for NHE measured in Hepes, or calculated from the difference between *J*_NHE+NBC_ and *J*_NBC_ measured in the presence of 5% CO_2_/HCO_3_^−^ buffer. *F*, flux analysis for NHE (calculated from difference between *J*_NHE+NBC_ and *J*_NBC_) in the presence or absence of 100 nm 14v (10–15 cells). *G*, flux analysis for NHE, derived from pH_i_ recovery in CO_2_/HCO_3_^−^-buffered Tyrode solution containing 15 μm S0859 (selective NBC inhibitor) in the presence or absence of 100 μm ATZ (10–15 cells).

The apparent lack of effect of CA on NHE in the native myocyte was investigated further by superfusing 14v, thus selectively removing CA_e_ activity. In CO_2_/HCO_3_^−^-buffered media, the DMA-sensitive component of flux was unaffected by CA_e_ inhibition (Fig. 4*F*), indicating that NHE activity was not significantly influenced by exofacial enzyme activity. The possible influence of CA_i_ on NHE was also explored by first inhibiting NBC activity in the presence of CO_2_/HCO_3_^−^ using the selective NBC antagonist S0859 (Ch'en *et al*. 2008), thus exposing NHE activity alone. Subsequent addition of membrane-permeant ATZ, which inhibits both CA_e_ and CA_i_, again had no effect on NHE activity (Fig. 4*G*). It thus appears that NHE activity in an intact ventricular myocyte is not enhanced by the presence of CA_i_ or CA_e_, even though these molecules can accelerate the hydration/dehydration kinetics of CO_2_/HCO_3_^−^ buffer.

### NBC activity not enhanced by CA_e_

The effect of CA_e_ on NBC activity was investigated by testing the effect of 14v on NBC activity after NHE had been pharmacologically inhibited with DMA or cariporide. In these experiments the superfusates contained 45 mm K^+^ to depolarize the membrane potential and enhance NBC activity (the dominant NBC isoform in heart is electrogenic (1Na^+^–2HCO_3_^−^) and thus voltage sensitive; Aiello *et al*. 1998; Yamamoto *et al*. 2005). As shown in the specimen records of Fig. 5*A*, 14v had no effect on the time course of NBC-mediated pH_i_ recovery from an acid load (induced by ammonium prepulse). As a result, the computed pH_i_ dependence of acid extrusion via NBC was unaffected (Fig. 5*B*). The result confirms that NBC activity in superfused myocytes is not catalysed by exofacial CA activity.

**Figure 5:**
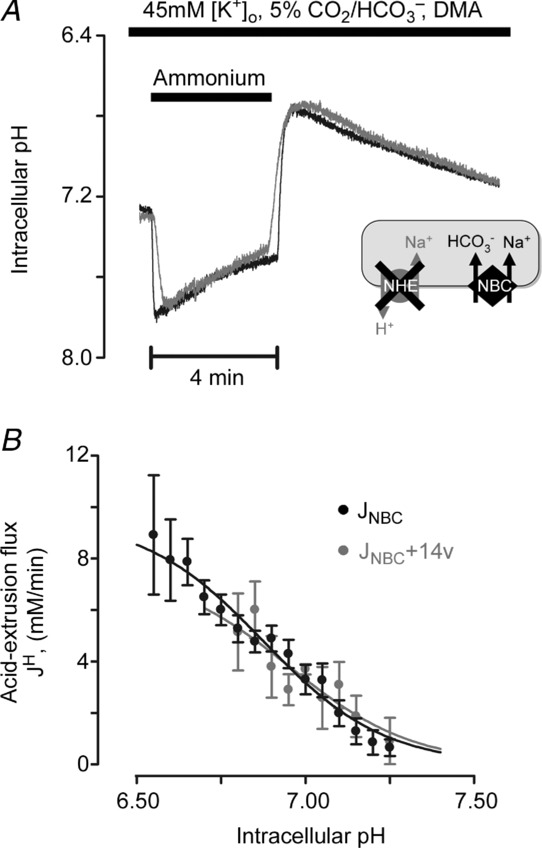
*A*, 20 mm ammonium prepulse solution manoeuvre in the presence of CO_2_/HCO_3_^−^, 30 μm DMA (NHE inhibitor) and 45 mm K^+^ (to increase electrochemical gradient for outward NBC current). Superimposed traces of NBC-mediated recovery of pH_i_ in the presence (grey) and absence (black) of 100 nm 14v (membrane-impermeant inhibitor). *B*, pH_i_ dependence of NBC flux (10–15 cells per data point).

### NBC activity enhanced catalytically by CA_i_

In contrast to the lack of effect of CA_e_ inhibition, there was a significant slowing of NBC activity when CA_i_ was inhibited. Figure 6*A* shows specimen records where addition of ATZ (or its analogue, ETZ) slowed NBC-mediated pH_i_ recovery from an intracellular acid load. In Fig. 6*B*, results have been averaged for cells superfused with Tyrode solution containing 4.5 mm K^+^ (Fig. 6*B* inset) or 45 mm K^+^ (to enhance NBC activity; Fig. 6*B* main panel). With both [K^+^]_o_ levels, generic NBC flux was halved in the presence of ATZ, although statistical significance was more evident over the pH_i_ range tested when NBC fluxes had initially been enhanced in high [K^+^]_o_.

**Figure 6:**
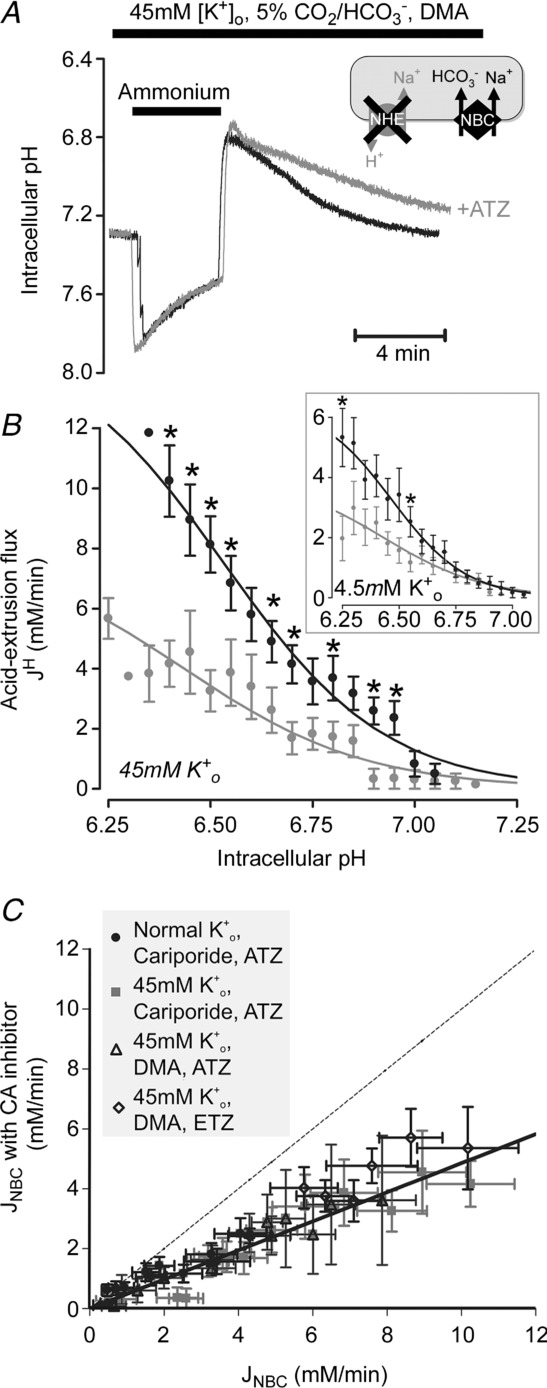
*A*, 20 mm ammonium prepulse solution manoeuvre in the presence of CO_2_/HCO_3_^−^, 30 μm DMA and 45 mm K^+^. Superimposed traces of NBC-mediated recovery of pH_i_ in the presence (grey) and absence (black) of 100 μm ATZ. *B*, pH_i_ dependence of NBC flux. ATZ caused significant reduction (51 ± 2%) of NBC flux (*n* = 3–12, *P < 0.05). Inset: pH_i_ dependence of NBC flux (*n* = 8–26; *P < 0.05). *C*, NBC-mediated acid efflux in the presence *versus* absence of CA inhibitor (100 μm ATZ or ethoxyzolamide, ETZ) at matching pH_i_, measured in 4.5 mm or 45 mm K^+^, in the presence of NHE inhibitors (30 μm DMA or cariporide). Identity shown as dotted line.

Figure 6*C* plots NBC flux measured at a given pH_i_ in the presence of ATZ or ETZ *versus* that measured in the absence of CA inhibition. Over almost all of the flux range measured, flux values fall well below the line of identity (dashed line, expected if flux is identical with and without ATZ or ETZ), indicating significant NBC slowing with CA inhibition. Given that selective CA_e_ inhibition exerted no effect, the slowing with membrane-permeant CA inhibitors is likely to be secondary to inhibition of CA_i_.

NBC slowing by ATZ was confirmed by measuring [Na^+^]_i_ changes associated with the activity of the transporter, as shown in Fig. 7. In this set of experiments, NBC was stimulated by a pH_i_ reduction induced with superfusion of 80 mm sodium acetate (salt of a membrane-permeant weak acid; cariporide was also added to inhibit NHE). During the activation of NBC, [Na^+^]_i_, recorded with intracellular SBFI (Na^+^ fluorophore, see Methods) increased. This [Na^+^]_i_ rise did not occur in the absence of CO_2_/HCO_3_^−^ buffer (*n* = 3, Hepes-buffered superfusates containing cariporide, not shown; see also Yamamoto *et al*. 2005), confirming that it was secondary to NBC activity. Addition of ATZ, which slows NBC-mediated pH_i_ recovery (Fig. 6), reduced the rate of rise of [Na^+^]_i_ by ∼40%, confirming attenuation of NBC flux.

**Figure 7:**
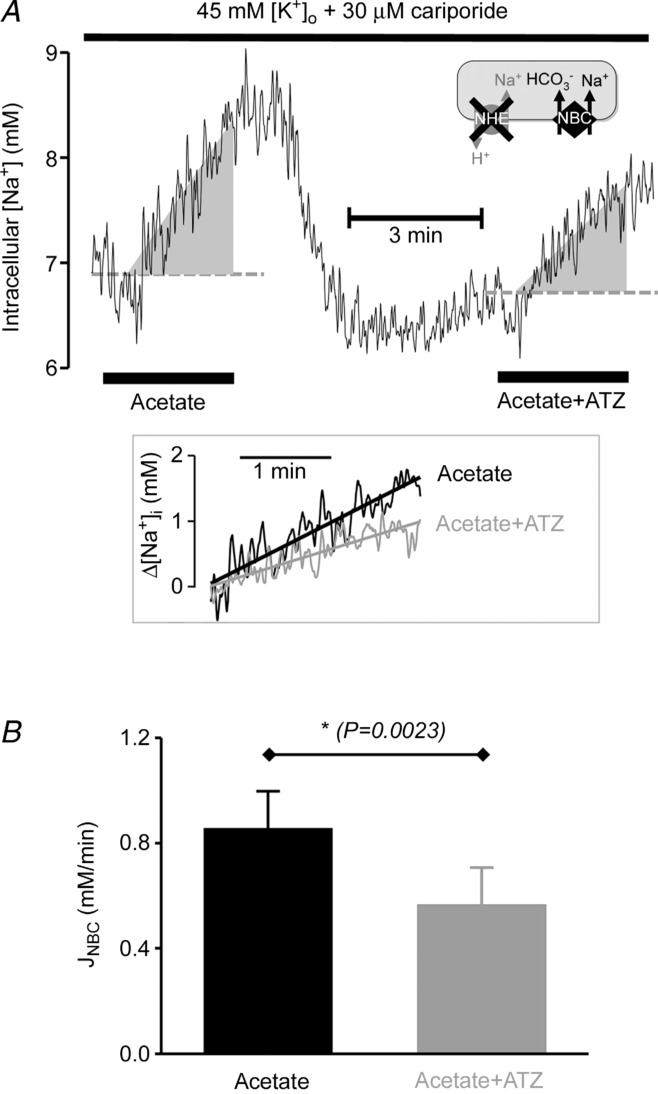
*A*, time course (average of 9 cells) of intracellular [Na^+^] ([Na^+^]_i_; AM-loaded SBFI fluorescence) calibrated using Na^+^ ionophores and pH_i_-corrected using pH_i_ template recorded in separate experiments (not shown; see Methods). Solutions contained 5% CO_2_/22 mm HCO_3_^−^ buffer, 45 mm K^+^ and 30 μm cariporide (NHE inhibitor). Na^+^ influx on NBC was stimulated by exposure to 80 mm sodium acetate (which rapidly reduces pH_i_). Paired experiment performed in presence of CA inhibitor ATZ (100 μm). *B*, rate of intracellular [Na^+^]_i_ rise, normalized to the starting level, gives a measure of NBC-mediated Na^+^ influx (*J*_NBC_). Initial rate was slower in ATZ (100 μm).

We conclude that NBC activity is enhanced by the catalytic activity of intracellular CA while NHE activity is not.

## Discussion

The present work provides the first demonstration in a native cell that CA activity selectively facilitates flux among pH regulatory transporters at the surface membrane. In the ventricular myocyte, selective facilitation relies exclusively upon intracellular CA, which enhances NBC activity, while exerting no effect on NHE1. In contrast, exofacial CA has no catalytic effect on either transporter. These results differ from previous work in heterologous expression systems where the flux activity of NHE1, NBCe1 and NBCn1, expressed in oocytes or various cell lines, has been suggested to be enhanced by the co-expression of either cytoplasmic CAII or exofacial CAIV (Li *et al*. 2002, 2006; Alvarez *et al*. 2003; Loiselle *et al*. 2004; Becker & Deitmer, 2007; Schueler *et al*. 2011), CA proteins that are endogenously expressed in the myocardium (Scheibe *et al*. 2006). It is notable that the spatial distribution of NBC (i.e. NBCe1 and NBCn1) and NHE1 protein is not identical at the surface membrane of the ventricular myocyte (Garciarena *et al*. 2013). NHE1 is expressed at intercalated disks and lateral sarcolemma, while NBC is particularly prominent in the transverse tubules (t-tubules). It is possible, therefore, that the facilitatory influence of CA_i_ activity on NBC flux is dependent on the local geometry at the transporter's expression site (this point is discussed later). Irrespective of the mechanism, however, by facilitating NBC activity in t-tubules, CA_i_ services pH_i_ regulation close to the machinery of excitation–contraction coupling, including L-type Ca^2+^ channels, ryanodine receptors, local sarcoplasmic reticulum Ca^2+^-ATPase pumps and Na^+^/Ca^2+^ exchangers, all of which are known to be pH sensitive.

### No role for exofacial CA in acid extrusion?

Several membrane-tethered CA isoforms with active sites oriented exofacially are expressed in mammalian ventricular myocytes. These include CAIV and CAXIV at the sarcolemma, with the additional possibility of CAIX in transverse tubules (Scheibe *et al*. 2006). The lack of a detectable influence of exofacial CA activity on H^+^ or HCO_3_^−^ transport in the present work is perhaps surprising, given that a stimulatory role has previously been proposed (Sterling *et al*. 2002; Alvarez *et al*. 2003), the enzymes collectively acting with pH_i_ regulatory proteins in what has been defined as a ‘transport metabolon’. This effect could represent the activity of a true metabolon, in which one or more exofacial CA molecules bind to the transporter to form a multimeric protein complex that displays enhanced H^+^-equivalent flux, or it could be a ‘functional metabolon’ that lacks direct protein–protein binding, but where a local sharing of common extracellular chemical substrates (e.g. H^+^ or HCO_3_^−^ ions) still enhances overall flux activity. The present work does not address the issue of protein–protein interactions, a phenomenon that is currently disputed (Boron, 2010; Al-Samir *et al*. 2013). The lack of enhanced NBC or NHE flux in the presence of CA_e_ suggests, however, that even a functional transport metabolon need not automatically result from co-expression of transporters and CA isoforms in the cell membrane. While this may indicate that the protein partners are not sufficiently co-localized in the native cell to result in shared functional activity, an additional possibility, as discussed below, is that experiments on isolated cells under superfusion may not reveal the full functional role of exofacial CA. Whatever the explanation, the present results show that there is no tight, obligatory link between exofacial CA expression and H^+^ or HCO_3_^−^ transporter activity in ventricular myocytes.

One explanation for the absence of CA_e_ interaction with ventricular NHE/NBC function relates to myocyte superfusion, an experimental procedure used in most cellular epifluorescence and imaging experiments. While superfusion provides controlled manipulation of the extracellular compartment, it also maintains the solution at the cell membrane close to CO_2_/HCO_3_^−^ equilibrium by continuously replacing it with freshly equilibrated Tyrode solution. Under these conditions, net CA_e_ activity, which accelerates carbonic buffer equilibration, will be close to zero, and thus unable to play a catalytic role in H^+^-equivalent transport (a similar phenomenon has been reported in cultured tumour cell lines; Swietach *et al*. 2008, 2009). The extracellular unstirred layer in superfused myocytes may therefore be sufficiently small and well coupled diffusively to the bulk solution such that local changes in extracellular pH, CO_2_ partial pressure and [HCO_3_^−^] are minimized during membrane acid/base transport. Although local pH_e_ changes do occur during high transmembrane H^+^-equivalent fluxes, and are regulated by exofacial CA activity (as shown in Fig. 3), the fact that CA_e_ inhibition does not influence overall NBC or NHE activity in these cells suggests that the changes are sufficiently small as to exert no significant feedback effect on transporter kinetics. This result does not exclude the possibility that important CA_e_ modulation of H^+^-equivalent transport might occur in multicellular myocardium, or even experimentally in clusters of NBC-expressing cells subjected to less efficient superfusion, where diffusive coupling in local extracellular spaces could be weaker. The key point is that potential coupling between CA_e_ expression and H^+^-equivalent flux in a given cell is not obligatory or invariant; any functional link will depend on the geometry of the local extracellular space and its diffusive linkage to the bulk phase.

### Catalytic role for intracellular CA in acid extrusion by NBC

While CA_e_ inhibition had no effect on acid extrusion through NBC, the membrane-permeant CA inhibitors ATZ and ETZ reduced NBC flux by up to 50% (Fig. 6), indicating a clear stimulatory role for the catalytic activity of *intracellular* CA (CA_i_). The native NBC isoform stimulated by CA_i_ cannot be identified in the present work. Both electrogenic (NBCe1) and electroneutral (NBCn1) isoforms are expressed in rat ventricular myocytes (De Giusti *et al*. 2011; Garciarena *et al*. 2013; NBCe2 transcripts have yet to be confirmed in rat myocytes), with electrogenic acid extrusion being a large flux component (Yamamoto *et al*. 2005). It is notable, therefore, that most previous work has found that the activity of both isoforms, when expressed heterologously in oocytes or cell lines appears enhanced by intracellular CA activity (Gross *et al*. 2002; Loiselle *et al*. 2004; Becker & Deitmer, 2007), although one report indicates no effect (Lu *et al*. 2006). The possibility that membrane-permeant sulphonamides interfere directly with NBC transport, rather than via inhibition of CA activity, is unlikely as ETZ had no effect on NBCe1 when expressed in oocytes in the absence of CA (Lu *et al*. 2006; Becker & Deitmer, 2007). The specific intracellular CA isoform involved in facilitating NBC flux is also not identified in the present work, but contributions are likely from cytosolic CAII as well as the membrane-tethered intracellular CAs identified in these cells, such as CAIV, XIV and IX, provided their active sites are oriented towards the cytoplasmic compartment (Scheibe *et al*. 2006; Schroeder *et al*. 2013).

A striking observation is that, while ATZ and ETZ slow NBC flux in the ventricular myocyte, they have no effect on NHE1, even in the presence of CO_2_/HCO_3_^−^ buffer (Fig. 4). This result rules out the possibility that the slowing of NBC-mediated pH_i_ recovery was caused by a general slowing of cytoplasmic CO_2_/HCO_3_^−^ buffering (subsequent to the inhibition of CA_i_), as this would have affected pH_i_ changes mediated by both types of transporter. Indeed mathematical modelling predicts no significant effect of slowing bulk cytoplasmic CO_2_/HCO_3_^−^ buffering with ATZ on the time course of NHE-mediated pH_i_ recovery (see Supplemental Fig. S1 available online). The slowing of NBC-mediated recovery must therefore represent a genuine reduction in membrane HCO_3_^−^ transport rate. The parallel reduction in the rate of rise of [Na^+^]_i_ associated with NBC activity (Fig. 7) provides independent corroboration of NBC inhibition, as do the results, presented below, of computational NBC modelling. The differential effect of CA_i_ on our estimates of NBC and NHE flux is therefore likely to represent a selective targeting by the enzyme of NBC's transport reactions. In the ventricular myocyte, CA_i_ and NBC appear to be partners in a ‘functional’ transport unit.

### Testing models of NBC facilitation by intracellular CA

#### Standard bicarbonate metabolon model does not predict flux facilitation

The classical explanation of CA_i_ facilitation of acid extrusion on NBC (largely by cytoplasmic CAII) is that the enzyme accelerates the reaction between H^+^ ions from bulk cytoplasm and HCO_3_^−^ ions unbinding from NBC, resulting in the formation of CO_2_ that escapes across the sarcolemma. This chemical reaction can be modelled using a simple mathematical function for pH_i_-sensitive NBC transport (where activity rises with a fall in pH_i_), in conjunction with the kinetics of HCO_3_^−^ protonation in the presence and absence of CA catalysis (measured experimentally, as shown in Figs 1 and 2). The model is presented schematically at the top of Fig. 8*A*, and full details are given in the Appendix. It predicts that even in the absence of catalytic CA_i_ activity, H^+^ titration of bulk intracellular HCO_3_^−^ proceeds sufficiently fast for it not to be rate-limiting for the overall NBC-driven acid-extrusion process, and hence will not significantly affect the overall rate of pH_i_ recovery. With spontaneous kinetics (rate constant *k*_r_ = 0.22 s^−1^ μm^−1^; Fig. 2*C*), constant 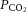 (5%), and a plentiful delivery of H^+^ ions from intrinsic buffers, the uncatalysed rate of HCO_3_^−^ protonation in cytoplasm (*k*_r_ × [H^+^] × [HCO_3_^−^]) will be ∼13 mm min^−1^, assuming the CO_2_/HCO_3_^−^ buffer remains close to equilibrium. This rate would be higher during the stimulation of NBC flux if intracellular [HCO_3_^−^] were to rise near the transporter, locally driving cytoplasmic CO_2_/HCO_3_^−^ buffer out of equilibrium. These spontaneous rates are in excess of the maximum measured flux on NBC (<12 mm min^−1^ in Fig. 6), indicating that the catalysis of bulk CO_2_/HCO_3_^−^ buffering by CA_i_ is not necessary to support transporter flux. Results of the computational modelling are shown in the main panel of Fig. 8*A*, which plots predicted NBC flux as a function of pH_i_, with and without CA_i_ activity (see also Supplemental Fig. S1). Both predictions are virtually coincident, indicating that this type of model fails to simulate the experimentally observed acceleration by CA_i_ of NBC flux. The classical explanation for a functional transport metabolon is thus not sufficient to account for CA facilitation of NBC.

**Figure 8:**
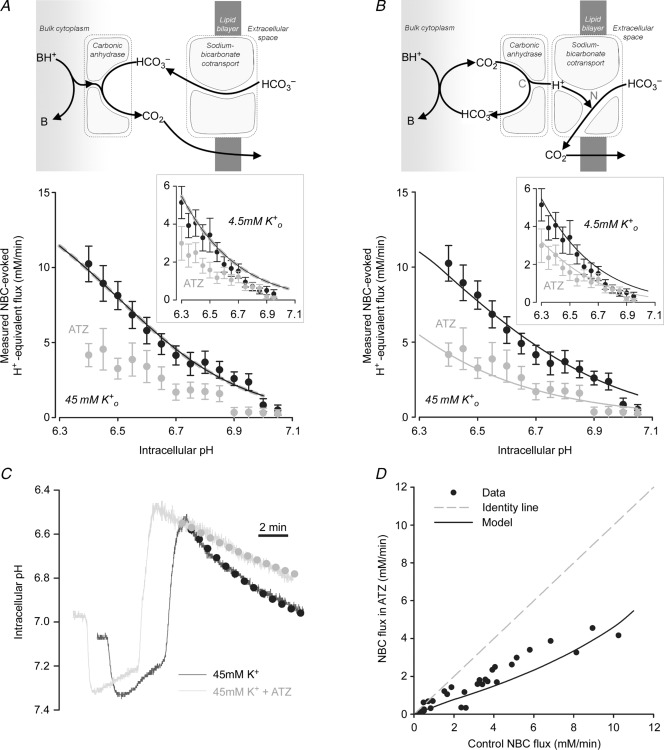
*A*, standard metabolon model. NBC imports highly mobile HCO_3_^−^ anion into the cytoplasm, where it reacts with H^+^ ions (supplied by intracellular buffers) to form CO_2_. This reaction can be catalysed by intracellular CA activity. NBC flux simulated by the model is unaffected by removing CA catalysis from the model (to simulate measured effect of ATZ). *B*, restricted H^+^ ion delivery model. HCO_3_^−^ anion, imported by NBC, reacts with H^+^ ion in a diffusionally restricted space (reaction N). Additionally, NBC activity is regulated allosterically by the pH in this restricted domain (rather than by the pH of bulk cytoplasm). Delivery of H^+^ ions directly from cytoplasm is rate limiting (e.g. because of binding to immobile titratable sites on proteins; quantified in terms of exchange rate constant *k*_slow_) but can be increased by CA activity (reaction C) providing H^+^ ions from catalytic CO_2_ hydration near the diffusionally restricted space. Best fit to data from Fig. 6*B* (45 mm K^+^ and 4.5 mm K^+^ in inset) for *k*_slow_ = 285 s^−1^. *C*, simulated time course of pH_i_ recovery (dotted lines) following an ammonium prepulse in the presence (grey) and absence (black) of ATZ, superimposed on experimental time courses (continuous traces). *D*, plot of NBC flux in the absence of CA inhibitors *vs*. NBC flux in the presence of ATZ (data points extracted from Fig. 6*C*), superimposed with model prediction (black line) and identity line (grey dashed line).

#### Restricted H^+^ ion delivery model predicts NBC facilitation by CA_i_

If, because of diffusional restrictions, local H^+^ ion *delivery* to the cytoplasmic face of NBC were rate-limiting, then the local HCO_3_^−^ protonation rate would be slowed (i.e. when *k*_r_ × [H^+^] × [HCO_3_^−^] < 13 mm min^−1^), which would reduce HCO_3_^−^ ion unbinding from the transporter and hence its kinetic turnover rate. If, in addition, the activity of NBC were regulated allosterically by a sensor that probed pH in such a diffusionally restricted domain, then NBC flux would be reduced further, because the protein would sense a pH that is locally more alkaline than in the bulk cytoplasm. Under these conditions, CA_i_ activity could facilitate NBC flux, by catalysing the local provision of H^+^ ions. The slowing of NBC observed with ATZ or ETZ is consistent with local H^+^ delivery arising from CA_i_'s catalytic activity (i.e. from CO_2_ hydration), as illustrated schematically at the top of Fig. 8*B*. In this model, H^+^ ions are delivered by CA_i_, but not by H^+^-carrying intrinsic mobile buffers, such as histidyl-dipeptides (Vaughan-Jones *et al*. 2002, 2009). These latter buffers are required, however, to supply the CA_i_ protein with H^+^ ions to regenerate CO_2_. Thus, the final H^+^ donation of the cytoplasmic H^+^ shuttle to NBC is made catalytically from a CA molecule. The donated H^+^ ion then combines spontaneously with HCO_3_^−^ within the NBC protein, resulting in the production of CO_2_ that escapes across the sarcolemma. On such a model, the H^+^ donation step from CA_i_ can become rate-limiting for NBC activity, as illustrated in the computational results shown in Fig. 8*B*. If CA catalysis is set to zero in the model (thus leaving only the slow rate of H^+^ generation from spontaneous CO_2_ hydration), then the pH_i_ dependence of HCO_3_^−^ influx on NBC (plotted in Fig. 8*B* as H^+^-equivalent extrusion) is shifted to lower flux levels (as also observed experimentally).

The kinetic model shown in Fig. 8*B* successfully predicts the time course of NBC-mediated pH_i_ recovery, and its slowing upon CA inhibition with ATZ or ETZ (Fig. 8*C*). The predicted reduction of NBC flux also matches experimental values over the whole flux range tested (Fig. 8*D*). The facilitation of NBC activity by intracellular CA is thus consistent with a functional transport unit, in which the enzyme catalytically donates H^+^ ions to the transport molecule rather than catalysing the subsequent HCO_3_^−^ titration reaction.

Free H^+^ ion diffusion in cytoplasm is negligible, because of high buffering by poorly mobile proteins (Vaughan-Jones *et al*. 2002; Swietach *et al*. 2007). Simple diffusion therefore cannot furnish an adequate H^+^ ion delivery to NBC. Small mobile buffers other than CA-catalysed carbonic buffer could potentially deliver the H^+^ ions. But, in this case, NBC activity would be unaffected by CA inhibition, which is contrary to experimental observation. The ability of CA_i_ to facilitate NBC activity therefore requires that local H^+^ ion delivery from intrinsic buffers be restricted. This might occur if the CA molecule acted as a gate to the NBC protein (see schematic diagram in Fig. 8*B*). For example, cytoskeletal geometry around the NBC and its local CA molecule might hamper intrinsic buffer access. As mentioned previously, NBC expression in the ventricular myocyte is prominent in the transverse tubules, close to L-type Ca^2+^ channels that communicate with the intracellular dyadic space (Garciarena *et al*. 2013). This is a crowded nanoscopic domain, ringed by structural as well as other Ca^2+^-handling proteins involved in excitation–contraction coupling. Alternatively, a physical barrier in the NBC transporter itself might restrict intrinsic buffer access, but permit a juxtaposition with CA_i_, thereby allowing the enzyme to donate H^+^ ions, while still allowing influx and efflux of CO_2_. Whatever the local geometry, following inhibition of CA_i_ activity with ATZ, spontaneous CO_2_ hydration would still appear to generate a supply of H^+^ ions that can fuel the lower uncatalysed rate of NBC activity (cf. Fig. 6 with Fig. 8*B*). The question of whether CA needs to be bound to NBC, as suggested by the proponents of a structural transport metabolon (Sterling *et al*. 2001, 2002; Morgan *et al*. 2007; Casey *et al*. 2009; Svastova *et al*. 2012) cannot be answered in the present work. A recent analysis, however, of CA-catalysed HCO_3_^−^ transport in the red blood cell (by the Band-3 Cl^−^/HCO_3_^−^ exchanger) suggests that a physical binding is unlikely (Al-Samir *et al*. 2013).

#### Restricted H^+^-ion diffusion model also predicts CA_i_ facilitation of a carbonate transporter

It should be emphasized that other kinetic models also predict CA_i_ facilitation of acid extrusion on NBC but, again, only when the enzyme catalyses H^+^ donation to the transporter, and contributions from intrinsic mobile buffers are greatly restricted (implemented in terms of a slow diffusive H^+^ ion exchange constant between bulk cytoplasm and the NBC transporter). For example, one alternative model (Boron, 2010) is that NBC may import carbonate (CO_3_^2−^) rather than HCO_3_^−^ anions, when operating in acid-extrusion mode (as argued experimentally for NBCe1; Zhu *et al*. 2013). In this model, CA_i_ activity could donate a H^+^ ion for the first protonation step, in order to release HCO_3_^−^ from the transporter and into the bulk cytoplasmic compartment, or donate H^+^ ions for both protonation steps, in order to release CO_2_ from the transporter. Such H^+^ donation, if catalysed by CA_i_, could again become rate-limiting for NBC activity. As a result, mathematical models of CO_3_^2−^ influx would predict enhancement of flux by CA_i_, providing the enzyme exclusively furnished the H^+^ donation. The predicted inhibitory effect of ATZ would be greater if CA_i_ were responsible for providing both H^+^ ions per CO_3_^2−^ transport cycle, rather than supplying the first protonation only. Indeed, any combination of H^+^ and HCO_3_^−^ membrane ion transport that encodes net acid efflux, can potentially be enhanced by CA_i_, as long as the enzyme donates H^+^ ions during transporter activity. Facilitation in all these models, however, still requires (i) ready H^+^ ion access to the transporter from active CA_i_, which implies relatively close proximity of the two proteins, and (ii) restricted H^+^ ion access to the transporter from intrinsic buffer shuttles, which would otherwise render CA_i_ activity redundant.

### Intracellular CA does not facilitate NHE1

The lack of facilitation of NHE1 by CO_2_/HCO_3_^−^ or CA_i_ in ventricular myocytes suggests that intracellular H^+^ ions delivered to this transporter are supplied from intrinsic (non-carbonic) buffers. As mentioned above, intrinsic H^+^ shuttling within cytoplasm is mediated by cytoplasmic mobile buffers, such as the histidyl dipeptides, carnosine, anserine and homocarnosine, which are collectively expressed at high concentration (∼15 mm) in ventricular myocytes (O'Dowd *et al*. 1988; Vaughan-Jones *et al*. 2002; Swietach *et al*. 2013). This H^+^ shuttle has a high diffusive capacity. Previous work in these cells has demonstrated that the shuttle can support longitudinal H^+^ fluxes of 50–100 mm min^−1^, while the CA-catalysed carbonic shuttle starts to saturate at rates above ∼8 mm min^−1^ (Swietach *et al*. 2005). These intracellular diffusive H^+^ fluxes should be compared with membrane transport fluxes stimulated at low pH_i_ on NHE1 (up to 25 mm min^−1^; Fig. 4*D*), and NBC (up to ∼12 mm min^−1^; Fig. 4*D*). The ATZ insensitivity and high capacity of acid extrusion through NHE1 therefore suggests that its H^+^ ion transport is supplied principally from the intrinsic dipeptide shuttle, while ATZ/ETZ sensitivity suggests that the terminal end, at least, of the cytoplasmic H^+^ shuttle to NBC is composed of a CA-catalysed delivery of H^+^ ions. These differing arrangements are illustrated schematically in Fig. 9. H^+^ ions from intrinsic mobile buffers thus appear to have ready access to the cytoplasmic face of NHE1 proteins. While this does not exclude delivery of CA_i_-derived H^+^ ions, the lack of inhibition of NHE1 flux by ATZ suggests the latter route must be small. The mechanism determining intrinsic buffer access to NHE1 but not NBCs is unknown. It may, for example, be related to the different spatial domains in which the two types of transporter are expressed in the ventricular myocyte.

**Figure 9:**
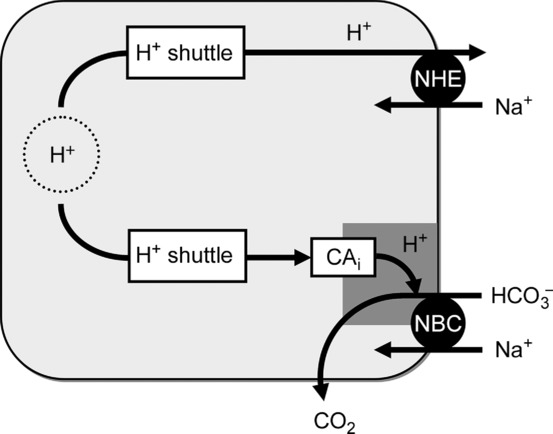
Schematic representation of proposed model. NBC: intracellular CA activity delivers H^+^ ions to a diffusionally restricted domain at the cytoplasmic surface of NBC protein, the H^+^ ions being necessary for the uncatalysed titration of its HCO_3_^−^ anion cargo. Reaction releases CO_2_, which diffuses freely across the myocyte and its surface membrane. The relevant CA_i_ is itself supplied with H^+^ ions derived from the bulk cytoplasmic H^+^ ion shuttle (composed mainly of mobile histidyl dipeptides). NHE: this transporter is not located in a diffusionally restricted space and can thus be supplied directly by H^+^ ions derived from the bulk cytoplasmic H^+^ ion shuttle.

Previous reports indicate that acid extrusion by NHE1 proteins, when over-expressed heterologously in *Xenopus* oocytes, is facilitated by CO_2_/HCO_3_^−^ (Li *et al*. 2002, 2006), provided intracellular CA (largely CAII) is also functionally active. Given the lack of CA_i_ facilitation in wild-type ventricular myocytes, this result is initially puzzling. One possibility is that intrinsic H^+^ ion mobility in oocytes is much lower than in myocytes, because of lower levels of diffusible intrinsic buffer. In the absence of an efficient intrinsic buffer shuttle, NHE transport will become more reliant on servicing by the intracellular carbonic buffer shuttle, and will thus be expected to display a more prominent facilitation by CA_i_ activity. This reliance of membrane flux on cytoplasmic H^+^ mobility may be exacerbated by the large size of the oocyte, resulting in unusually long diffusion delays for H^+^ ions from the cytoplasmic core to NHE transporters at the periphery, delays that may be lengthened further by cytoplasmic CA inhibition. Whatever the cause of CA_i_ facilitation of NHE1 in oocytes, the present work shows clearly that CA_i_ activity is not necessary for physiological NHE1 function in the native ventricular myocyte.

### Conclusions

We conclude that, in the native ventricular myocyte, intracellular CA enzymes form a functional transport unit with NBC, thereby enhancing its flux. Our results disagree in important respects with several previous reports of a HCO_3_^−^ transport metabolon, based on heterologous expression systems. Although catalytic CA_i_ activity appears to enhance NBC flux in expression systems and wild-type cardiac cells, the native cardiac transporter is insensitive to extracellular (exofacial) CA activity. In addition, native cardiac NHE1 activity is entirely CA insensitive. The coupling of CA_i_ to NBC flux in myocytes does not necessarily imply physical binding between the two molecules (a classical transport metabolon), a non-covalent juxtaposition may suffice, with the proteins interacting functionally. A novel finding is that CA_i_ facilitation of NBC is quantitatively consistent with the enzyme catalysing the *delivery* of H^+^ ions to the cytoplasmic face of the transporter, rather than the subsequent protonation of the HCO_3_^−^ substrate. In contrast, the lack of modulation of NHE1 flux by CA_i_ suggests that, in the ventricular cell, this particular transporter is adequately supplied with H^+^ ions via intrinsic mobile buffers. CA_i_ activity therefore plays a key role in regulating native NBC but not NHE activity in cardiac cells (Fig. 9). By spatially trading H^+^ ions, the myocyte's intracellular CA enzymes behave as proton-coupling proteins, linking pH_i_ to membrane HCO_3_^−^ transport, in order to stabilize [H^+^]_i_ levels and thus physiological function in the heart.

## Key points

- Carbonic anhydrase enzymes are said to enhance membrane H^+^ and HCO_3_^−^ transport, an idea tested almost exclusively in heterologous cell-expression systems.
- In ventricular myocytes, inhibiting cytoplasmic enzyme activity slows membrane acid extrusion (HCO_3_^−^ influx) on Na^+^–HCO_3_^−^ co-transporters, with no effect on Na^+^/H^+^ exchangers. Inhibiting exofacial enzyme activity has no effect on either transporter.
- Mathematical modelling simulates the influence of carbonic anhydrase on Na^+^–HCO_3_^−^ co-transport, provided the enzyme catalytically *delivers* H^+^ ions to the transporter (across a cytoplasmic nanodomain that is poorly accessible to intrinsic buffers), rather than catalysing protonation of the imported bicarbonate. Intrinsic cytoplasmic mobile buffers appear to deliver H^+^ to Na^+^/H^+^ exchangers, thus obviating the need for carbonic anhydrase.
- Conclusion: in native cardiac cells, intracellular carbonic anhydrase molecules partner Na^+^–HCO_3_^−^ co-transporters but not Na^+^/H^+^ exchangers, to enhance flux activity. The enzyme thus plays a key role in supporting bicarbonate transport and pH control in the heart.

## Appendix

### Appendix

A mathematical model was developed to explore the effect of carbonic anhydrase (CA) catalytic activity on pH_i_ regulation by NBC flux (*J*^NBC^). The transporter flux was modelled using the Hill equation (Yamamoto *et al*. 2005):

where [H^+^]_c_ represents cytoplasmic [H^+^]. In comparison to *J*^NBC^, CO_2_ fluxes are not rate-limiting because of rapid membrane permeability and diffusivity across all cell compartments. To simplify the equations, CO_2_ levels were clamped (d[CO_2_]/d*t *= 0) at 1.2 mm. Table 1 lists the parameters of the model.

**Table:** Variables used in the mathematical models (see Appendix)

Parameter	Symbol	Value	Reference
Intrinsic buffering capacity	β_int_	(a function of pH_i_)	Zaniboni *et al*. (2003)
Myocyte length	*L*	120 μm	Present work
Myocyte radius	*R*	10 μm	Present work
NBC maximal flux (basal)	*V*_max_	6.5 mm min^−1^	Fig. 6*B*
NBC maximal flux (stimulated)	*V*_max_	22 mm min^−1^	Fig. 6*B*
NBC internal H^+^ affinity constant	*K*_m_	10^−6.5^ m	Fig. 6*B*
NBC Hill co-operativity for internal H^+^ ions	*n*	2 (unitless)	Fig. 6*B*
Cytoplasm-averaged carbonic anhydrase activity	CA	3 (unitless)	Fig. 2*C*
Spontaneous CO_2_ hydration constant	*k*_f_	0.18 s^−1^	Fig. 2*C*
Spontaneous HCO_3_^−^ dehydration constant	*k*_r_	0.22 μm^−1^ s^−1^	Fig. 2*C*
CO_2_ acid-dissociation constant	*K*	10^−6.1^ m	Leem & Vaughan-Jones (1998)
Rapid transfer constant between compartments	*k*_rapid_	>10^4^ s^−1^	Best fit
Slow transfer constant between compartments	*k*_slow_	250 s^−1^	Best fit

#### Standard metabolon model

The first model was based on the scheme shown in Fig. 8*A*. The equations for cytoplasmic [HCO_3_^−^] ([HCO_3_^−^]_c_) and [H^+^]_c_ were:

where CA is a measure of carbonic anhydrase activity and β_int_ is the intrinsic buffering capacity in units of mm (pH unit)^–1^ (converted to dimensionless buffer ratio by dividing by 2303 × [H^+^]).

#### H^+^ ion-limited model

In the second model (Fig. 8*B*), additional equations were included to simulate the behaviour of [HCO_3_^−^] and [H^+^] in a sub-membrane diffusionally restricted (unstirred) domain ([HCO_3_^−^]_u_, [H^+^]_u_) and a transporter-associated CA domain ([HCO_3_^−^]_ca_, [H^+^]_ca_):

In this model, flux *J*^NBC^ is a function of [H^+^] in the unstirred layer. The volume fractions of the cytoplasmic, unstirred and carbonic anhydrase domains were given by constants *F*_c_, *F*_u_ and *F*_ca_. The bulk cytoplasm was assumed to be, by far, the largest (*F*_c_ ≈ 1). Diffusive transfer of ions between compartments was modelled with a rapid or slow (rate-limiting) exchange rate constant, *k*_rapid_ or *k*_slow_, respectively. H^+^ ions exchanged rapidly between the CA and unstirred domains, but only slowly between the cytoplasmic and unstirred domains. The experimentally observed CA dependence of NBC activity might be explained by allowing a direct H^+^ ion delivery route from the CA catalytic site to the transporter protein. The basis for this H^+^-shuttling mechanism may be the recently proposed H^+^-collecting antenna in CAII (Becker *et al*. 2011). To simulate this privileged H^+^-shuttling pathway, diffusive exchange of H^+^ ions was allowed to be fast between CA and NBC, but negligible between CA and the bulk cytoplasm. HCO_3_^−^ ions were permitted to exchange freely between the cytoplasmic and CA domains, but not between the unstirred and cytoplasmic domains (a condition that is expected if HCO_3_^−^ ions reacted to form CO_2_ faster than they would unbind from the transporter). To simulate pharmacological CA inhibition, reactions in the CA domain were set to zero and hydration kinetics in the cytoplasmic domain were set to spontaneous levels (NB: reactions in the unstirred domain proceed at spontaneous kinetics irrespective of the presence or absence of ATZ). The inhibitory effect of ATZ or ETZ on NBC flux could be simulated mathematically by setting *k*_slow_ to a rate-limiting value (relative to *k*_rapid_). By selecting an adequately large *k*_rapid_, the transfer of H^+^ and HCO_3_^−^ between the cytoplasmic and CA domains becomes non-rate-limiting. The results of the simulation are therefore not sensitive to the values for *F*_ca_ and *F*_u_, provided these are small. The best fit to the experimental data (i.e. a ∼50% decrease in NBC flux in the presence of ATZ) could be simulated for *k*_slow_ = 285 s^−1^ (*k*_rapid_ > 10^5^ s^−1^; *F*_u_ > 10^−6^, *F*_ca_ > 10^−6^). For comparison, the Ca^2+^ diffusion exchange constant between the cytoplasm and subsarcolemmal fuzzy space is 2500 s^−1^ (Snyder *et al*. 2000). The value of *k*_slow_ is related to H^+^ ion diffusivity, *D*_H_, and diffusion distance δ between the cytoplasmic and unstirred domains:

H^+^ ion diffusivity in water is 1.2 × 10^4^ μm^2^ s^–1^, and this value yields a distance δ = 7 μm, which is unrealistic for the geometry of the myocyte. However, H^+^ ion diffusivity is reduced due to binding with fixed titratable sites (such as those presented by proteins). If the ratio of bound to free H^+^ ions is *B*, diffusivity will be reduced by a factor of (*B* + 1), assuming that none of the buffers is mobile in the unstirred domain. A further reduction is expected due to volume exclusion by macromolecules. For a volume exclusion of 50% and an intracellular buffering capacity of 30 mm (pH unit)^–1^ (*B* = 2 × 10^5^), distance δ is predicted to be 10 nm, i.e. comparable to the width of a lipid bilayer.

The model above assumes that the transported substrate is HCO_3_^−^. If, instead, the transporter protein handled CO_3_^2−^ ions, as proposed recently (Boron, 2010; Zhu *et al*. 2013), then an additional protonation step would be required in the transport cycle. The diffusive barrier to cytoplasmic H^+^ delivery could apply to the first (H^+^ + CO_3_^2−^ → HCO_3_^−^), second (H^+^ + HCO_3_^−^ → CO_2_) or both protonation steps. If both protonation steps were subject to the diffusive barrier, the best-fitting distance δ would be smaller.

## Abbreviations

ATZ: acetazolamide
CA: carbonic anhydrase
CA_i_: intracellular CA
CA_e_: extracellular exofacial CA
DMA: dimethylamiloride
ETZ: ethoxyzolamide
NBC: Na^+^–HCO_3_^−^ cotransport
NHE: Na^+^/H^+^ exchange
WGA-fluorescein: wheat germ agglutinin-conjugated fluorescein
