# Supplementary material for: Facilitation by intracellular carbonic anhydrase of Na+–HCO3− co-transport but not Na+/H+ exchange activity in the mammalian ventricular myocyte
Source: J Physiol. 2014 Jan 9;592(Pt 5):991–1007. doi: 10.1113/jphysiol.2013.265439 (PMC3948559; doi:10.1113/jphysiol.2013.265439)
Supplement: Supplementary file 1 [file tjp0592-0991-sd1.docx]

| A. |  |
| --- | --- |
| B. |  |

**Supplemental Figure S1. *Mathematical simulation of the effect of CA_i_ activity on NHE flux.*** NHE activity was modelled using Hill equation (V_max_=30 mM/min; K=10^-6.75^ M, Hill coefficient 2). Intrinsic and carbonic buffering was present. In the presence of CA_i_ activity, the CO_2_ hydration constant k_f_ was 0.54 s^−1^; in the absence of CAi activity, kf was 0.18 s^−1^. Acid-efflux was calculated from the simulated time course of pH_i_ recovery from an acid-load (pH_i_=6.2). Flux calculated from rate of pH_i_ change × buffering capacity. (A). Buffering capacity assumed to be the sum of intrinsic buffering (measured previously) and carbonic buffering at equilibrium (2.303×[HCO_3_^−^]_e_×10^pHi-pHe^) (B). Buffering capacity assumed to be the sum of intrinsic buffering (measured previously) and carbonic buffering derived from the simulated time course of intracellular [HCO_3_^−^] (2.303×[HCO_3_^−^]_i_), instead of assuming trans-membrane equilibrium.
